# Development and outcomes of surgical and urological kidney transplantation programs in Germany: a total population analysis from 2006 to 2021

**DOI:** 10.1007/s00345-023-04740-1

**Published:** 2024-02-01

**Authors:** Philipp Reimold, Cem Aksoy, Jonas Beckmann, Aristeidis Zacharis, Christer Groeben, Philipp Karschuck, Nicole Eisenmenger, Josef Geks, Johannes Huber, Luka Flegar

**Affiliations:** 1 Department of Urology, Philipps-University Marburg, Marburg, Germany; 2 Reimbursement Institute, Hürth, Germany; 3 Department of Surgery, Philipps-University Marburg, Marburg, Germany

**Keywords:** Kidney transplantation, Urology, Surgery, Outcomes, Health services research

## Abstract

**Purpose:**

Kidney transplantation (KT) is the most frequently performed organ transplantation. In Germany, KT is performed in urology and surgery departments with unknown consequences of this parallel structure. The aim of the study was to compare the development and outcome of KT in urology and surgery departments.

**Methods:**

On an institutional level, we analyzed the annual caseload from 2006 to 2021 with the reimbursement. INFO tool based on hospitals’ quality reports (Reimbursement Institute, Hürth, Germany). For outcome comparison we extracted raw data from the transplantation centers' quality reports (Deutsche Stiftung Organtransplantation, DSO).

**Results:**

A total of 23,599 cases (17,781 deceased donor and 5,818 living donor KTs) were included. The total number of KTs decreased from 1851 in 2006 to 1701 in 2021 (− 8%; p = 0.12). The total number of urological KTs decreased from 592 cases in 2006 to 395 cases in 2021 (− 33.3%; *p* = 0.01). Further analysis revealed no significant differences between intra- and postoperative complications and graft quality at one year for deceased donor KTs (DDKT) although differences in immediate renal function and graft quality at discharge could be observed. There were no significant differences in immediate renal function and graft quality at discharge for living donor KTs (LDKT) between the specialties.

**Conclusion:**

KTs performed in urology departments declined between 2006 and 2021. Nevertheless, intra- and postoperative complications as well as long-term function did not differ between surgical and urological KT programs. Hence, an interdisciplinary approach, especially considering the upcoming challenges in KT as, e.g., robot-assisted surgery seems reasonable.

**Supplementary Information:**

The online version contains supplementary material available at 10.1007/s00345-023-04740-1.

## Introduction

Kidney transplantation (KT) remains the best modality of renal replacement therapy for patients with end-stage renal disease. There is an ever-increasing demand for organ donation, especially given that the global burden of chronic kidney disease is increasing and it is projected to become the fifth most common cause of years of life loss globally by 2040 [1].

Traditionally, urologists have been involved in KTs since the first KT was successfully performed on two genetically identical twins by the surgeon Joseph Murray (recipient’s transplantation) and the urologist Hartwell Harrison (living donor nephrectomy) in 1954 [2]. The first clinically relevant KTs in Germany were performed by the urologists Brosig and Nagel in Berlin in 1963 [3]. Since then, KTs in Germany have been conducted partly in departments of urology and partly in departments of surgery.

Organs can be donated either from deceased donors (deceased donor kidney transplantation, DDKT) or living donors (living donor kidney transplantation, LDKT) suitable for transplantation. Germany ranks low among the Eurotransplant countries for decades and is in 35th place in a global comparison [4]. Nevertheless, various efforts to improve the procedure have been made recently especially in the context of robot-assisted KT [5].

While urologists and surgeons have different focuses and unique characteristics, both disciplines have historically run KT programs in Germany. However, this historically grown structure has not been studied so far to identify potential differences in performance and outcomes. The aim of the present study was to compare and assess the performance and complication rates between urological and surgical transplant centers. A better understanding of these factors within the German landscape of KT can significantly contribute to improving clinical decision-making and enhancing patient care in the future.

## Patients and methods

### Databases

For this study, we used two datasets. We analyzed data from German hospitals’ quality reports as well as from the activity reports of the German Organ Procurement Organization (Deutsche Stiftung Organtransplantation, DSO).

The German hospitals’ quality reports were used for identification of national providers. We described the data extraction and cohort identification methods in previous studies [7]. On an institutional level, we analyzed the annual caseload with the reimbursement. INFO tool (Reimbursement Institute, Hürth, Germany) based on hospitals’ quality reports. Approximately, 85–90% of the cases from the hospitals’ quality reports are represented in the dataset of the DSO.

KTs were defined by OPS (Operationen- und Prozedurenschlüssel) code 5–555.0 (LDKT) and code 5–555.1 (DDKT). Departments were classified by the department code (FAB − Fachabteilungsschlüssel: urology 2200 and surgery 1500, 1518, 1520 and 1550). We defined departments with > 25 KTs and > 5 LDKTs as high-load transplant centers corresponding to the present cutoff for certified centers in Germany. Maps were rendered by using the software “EasyMap 11.1 Standard Edition” (Lutum + Tappert DV-Beratung GmbH, Bonn, Germany).

We queried the activity reports from the DSO for intra- and postoperative complications, graft function, graft quality at discharge and graft quality at one year. The reports were downloaded from the DSO Website (https://dso.de/organspende/statistiken-berichte/berichte-der-transplantationszentren) and screened for complete datasets. Complete datasets could be analyzed for the years 2013–2019 (complications, overall good graft quality at discharge) and 2013–2020 (immediate function after transplantation), respectively. Due to incomplete reports for the other years, the rate of good graft quality after one year could only be analyzed for DDKTs between 2015 and 2019.

Intra- and postoperative complications in KT in the dataset of the DSO are defined as severe complications leading to blood transfusion or revision surgery. The indications for revision surgery are not reported in the quality report of the DSO. Immediate function was defined as a maximum of one postoperative dialysis until the day of discharge. The overall good quality of the transplanted organs was defined as GFR ≥ 20ml/min.

### Statistical analysis

Data were presented by absolute and relative frequencies. To detect trends over time linear regression models were implemented. Chi2-test was performed to compare the relative rate of events between the groups. We defined p < 0.05 to indicate statistical significance. We used SPSS 28.0.1.1. (IBM corp., Armonk, NY, USA) for our statistical analysis.

### Ethics statement

We conducted this study in accordance with the Declaration of Helsinki in its latest version. No further ethics committee approval was required because we only analyzed anonymous aggregated data from established databases. Further, a written informed consent was not needed. For data protection reasons, within the quality reports, the diagnostics (ICD) data or intervention numbers (OPS) with a number of ≤ 3 do not indicate the actual number, but the number 1.

## Results

### Caseload analysis of urological and surgical KT programs

**Fig. 1:**
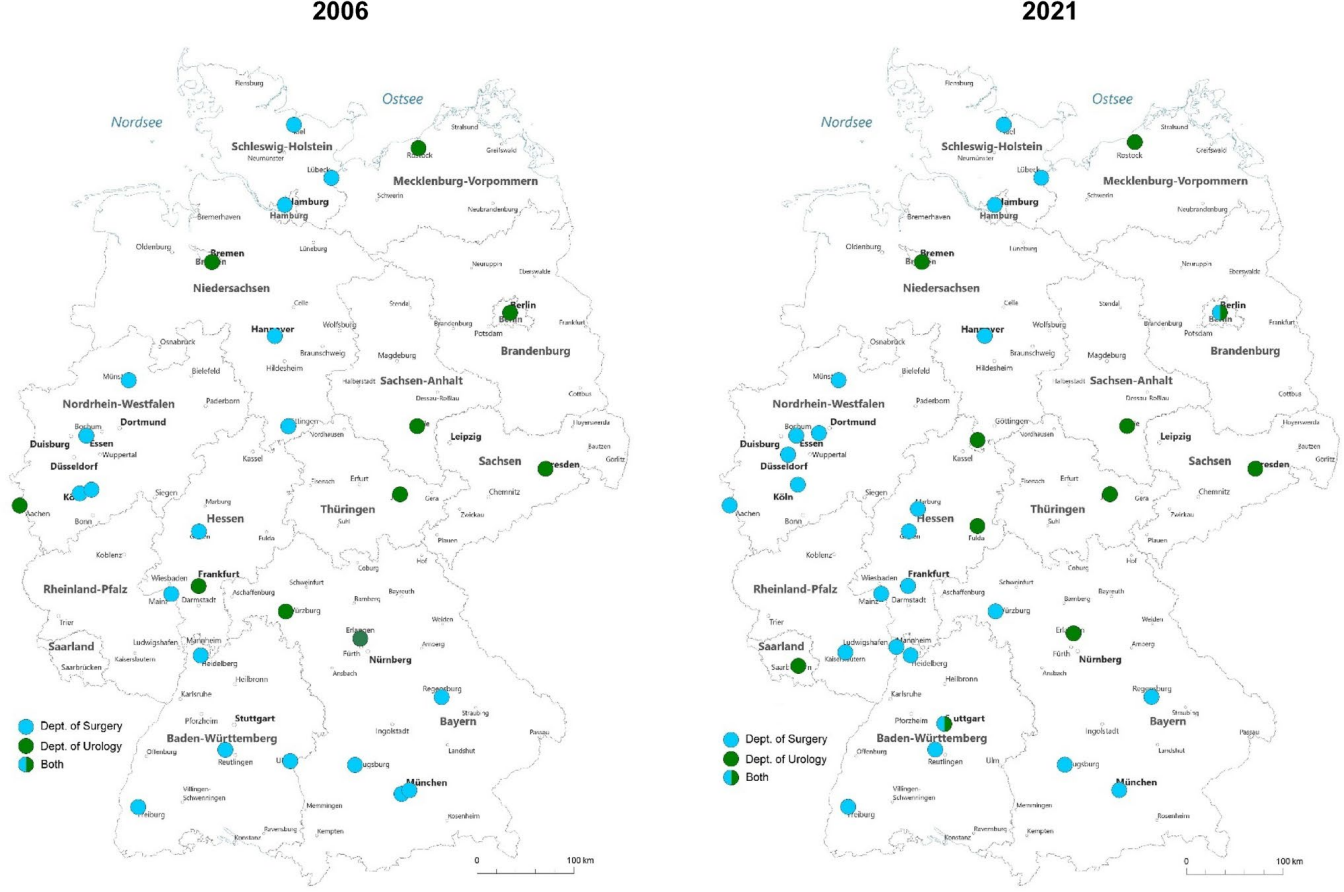
Departments of Surgery and Urology performing KTs in 2006 (left) and 2021 (right)

**Fig. 2:**
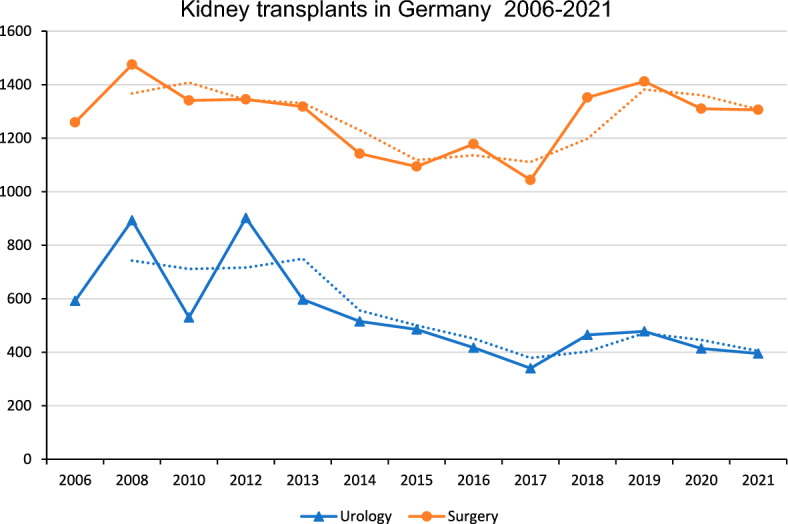
Kidney transplants in Germany from 2006 to 2021 based on the hospitals’ quality reports (extracted with the reimbursement.info tool)

We included a total of KT 23,599 cases, thereof 17,781 (75.3%) DDKTs and 5,818 (24.7%) LDKTs. The total number of KTs decreased from 1851 in 2006 to 1701 in 2021 (− 8%; *p* = 0.12). In 2006, 10 urological and 19 visceral surgery hospitals performed KTs. In 2021, 11 urological and 25 visceral surgery clinics performed KTs. The landscape of centers performing KT in 2006 and 2021 is shown in Fig. 1. The total annual number of urological KTs decreased from 592 cases in 2006 to 395 cases in 2021 (− 33.3%; *p* = 0.01). The total annual number KTs performed in visceral surgery departments was 1,259 cases in 2006 and remained constant at 1306 cases in 2021 (+ 3.7%; *p* = 0.59, see Fig. 2). The proportion of urology departments with < 25 KTs/year increased from 33 to 42%, while for visceral surgery departments it increased from 26 to 37%. The proportion of urology clinics with < 5 LDKTs/year remained constant at 33% between 2006 and 2021 and increased from 20% in 2006 to 24% in 2021 for surgery departments. The regional distribution of the departments of surgery and urology performing KTs is shown in Fig. 1, exemplarily for 2006 and 2021.

### Outcome analysis of urological and surgical KT programs

**Fig. 3:**
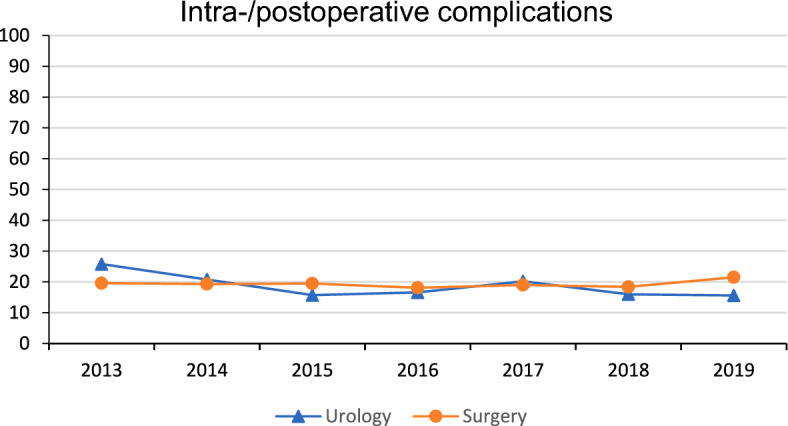
Intra- and postoperative complications in deceased donor kidney transplants

Between 2013 and 2019, the rate of complications for DDKT did stay around 19% in urologic and surgery departments (mean 18.6%; SD 3.5% vs. mean 19.3%; SD 1.0%; *p* = 0.404) and did not differ significantly (see Fig. 3, Suppl. Table 1).

Between 2013 and 2020, the rate of transplants with immediate function after transplantation did differ significantly in DDKTs. A mean of 72.3% (SD 2.8%) of the organs transplanted in urologic departments and 80.1% (SD 3.9%) of the organs in surgical departments showed immediate function (*p* < 0.001). For LDKT grafts in urologic departments, 96.0% (SD 2.2%) and 97.1% (SD 1.7%) of grafts in transplanted in surgical departments showed immediate function (*p* = 0.065). The detailed comparison of all the years reported are shown in Suppl. Fig. 1 and Suppl. Table 2.

In the years 2013–2019 no significant difference in graft quality at the day of discharge was reported in LDKT (urology: 96.4%, SD 1.2%; surgery: 95.8%, SD 1.0%; *p* = 0.404). For DDKT, a significant difference in graft quality can be reported for the years 2013–2018 (urology: 84.0%, SD 4.6%; surgery: 85.8%, SD 2.4%; *p* = 0.037; Suppl. Fig. 2, Suppl. Table 3).

In addition to the short-term data for immediate function and quality of the transplanted kidneys, long-term data for the quality of the grafts one year after surgery are shown in Suppl. Fig. 3, Suppl. Table 4. Mean long-term kidney function was 96.3% (SD 0.9%) for urology and 96.1% (SD 0.8%) for surgery (*p* = 0.602).

## Discussion

Our study describes the development and distribution of the urology and surgery departments performing KTs in Germany between 2006 and 2021. There is a decline in overall KT numbers as well as the share of procedures conducted in urology departments. Intra- and postoperative complications occur in about 19% of the cases between 2013 and 2019, independently of the treating specialty. Furthermore, we show that intra- and postoperative complications and graft quality at one year for DDKTs did not differ significantly between urology and surgery departments, although differences in immediate renal function and graft quality at discharge could be observed. There were no significant differences in immediate renal function and graft quality at discharge for LDKTs between the specialties. These findings show good quality of the German transplantation departments regardless of the specialty being in charge.

The reported decrease in KTs in our dataset from 1851 in 2006 to 1701 in 2021 (− 8%) is in contrast with European data showing an increase from 18,490 in 2010 to 21,235 in 2019 (+ 14.8%) [8]. As the distribution of organs is organized in a centralized manner for every member country in the Eurotransplant program, this declining trend in KTs in Germany is most likely due to the national structure of postmortal organ donations. The organ donation rate in Germany is low—in 2022 only 869 postmortal donations were registered [9]. Compared to 2021 it decreased around 7% [10]. The rate is proposed to decline due to a recognition and reporting deficit of potential organ donors [11]. In addition to that, a lot of trust in the institutions was lost during the German transplantation scandal in 2012, potentially having had a negative impact on the donor rate, as well [12, 13]. Furthermore, donation after circulatory death (DCD) as a concept to improve organ donation rate, is not performed in Germany in contrast to other European countries [14].

Regarding complication rates, this study is in line with data reporting overall surgical complications in up to 20% of the recipients [15, 16]. It is important to consider, that reports about complication rates are mostly based on single-center reports and are not compared between the surgical specialties.

Immediate postoperative organ function in DDKTs did differ significantly between urology and surgery departments in our analysis. For DDKT, a mean of 72.3% (urology) and 80.1% (surgery) of the organs showed immediate function (*p* < 0.001), whereas for LDKT the rate was 96.0% in urology and 97.1% in surgery (*p* = 0.065). Our data are in line with reports that show delayed graft function in 24% of kidney recipients in the United States in 2021 [17, 18]. The rate of delayed graft function in LDKT is comparable as well, reaching up to 4.9% in centers [18, 19].

The data analyzed in the present study was extracted from the activity reports of the DSO. Participating hospitals in external comparative quality assurance must report case numbers and documentation rates for the relevant services. Complete documentation is required, although errors can occur, leading to potentially inaccurate data.

Especially in low-volume centers, data accuracy of the quality reports was low. Furthermore, we observed differences in the reported total numbers of performed procedures when comparing data from the DSO with extracted data from the reimbursement.info tool. Approximately 85–90% of the cases appear to be represented in the data from DSO’s quality reports.

The COVID-19 pandemic has been linked to incomplete documentation impacting healthcare services, including transplantation programs globally [21, 22]. Notably, quality indicator results are influenced not just by hospitals but also by factors like concomitant diseases, disease severity, and patient age. Regional disparities in patient care have also been identified as a factor in transplantation [3].

Intra- and postoperative complications are defined as severe complications leading to blood transfusion or revision surgery in the data of the DSO. Of course, the landscape of complications during or after KT is more complex. When comparing urology and surgery departments, one also must compare specialty-specific complications. The most relevant complications can be divided in vascular complications, complications of the urinary tract and lymphoceles [23]. Vascular complications, e.g., renal artery or vein thrombosis, iliac artery dissection or renal artery stenosis have an incidence of 0.8–6% [24]; whereas, urologic complications such as urine leakage or ureteral obstruction occur in 2.5–30% of all recipients [23]. The likelihood of complications increases with lower quality of the offered organs and comorbidities of the recipients. In the datasets analyzed, no information about graft quality (e.g., ischemia time) and patients’ comorbidities or age (e.g., rate of expanded criteria donors) was given, so we cannot rule out whether the performance of certain centers was influenced by their selection of organs and recipients.

Due to the structure of the quality reports provided by the DSO a detailed analysis of intra- and postoperative complications was not possible. However, since these are the only epidemiological data for Germany, we had to accept this limitation.

The quality of the transplanted organs at the day of discharge was defined as good at a GFR ≥ 20 ml/min in this dataset. This makes a comparison to other publications difficult as they mostly report early and delayed graft function by defining a dialysis within the first 7 days as cutoff. Likewise, the one year quality as defined in our dataset is not comparable to other studies in the field, as the range of GFR ≥ 20 ml/min is too wide. An analysis of an American cohort showed 1 year post transplant GFR around 57 ml/min per 1.73 m^2^ [20].

To address the question of specific complications in urology and surgery departments, further research is needed. The hypothesis could be, that urology departments have a lower incidence of urinary tract complications and surgery departments have a lower vascular complication rate. A prospective multicenter design would be a suitable approach.

The aim of future advances in transplantation surgery should be to decrease morbidity of the procedure and achieve the highest quality possible. An interdisciplinary approach seems reasonable and has been reported in the past to improve outcomes of transplantations [25, 26]. Especially recent advances in robot-assisted KT could serve to improve overall quality of the procedure. Robot-assisted KT is emerging in centers in Germany and Europe and has been shown to be at least non-inferior to the open procedure [6, 27].

## Conclusion

Despite decreasing numbers of kidney transplantations in Germany, very good short- and long-term results can be achieved regardless of the fact whether the procedure is performed in a department of urology or a department of surgery. A detailed analysis of both intra- and postoperative complications, affecting approximately 19% of the cases is needed to improve clinical decision-making and patient care.

## Supplementary Information

Below is the link to the electronic supplementary material.

Supplementary file1 (DOCX 18 KB)

Supplementary file2 (DOCX 17 KB)

Supplementary file3 (DOCX 15 KB)

Supplementary file4 (DOCX 20 KB)

## Data Availability

The datasets used and analyzed during the current study are available from the corresponding author on reasonable request.

## Abbreviations:

DCD: Donation after circulatory death
DDKT: Deceased donor kidney transplantation
DSO: Deutsche Stiftung Organtransplantation
GFR: Glomerular filtration rate
KT: Kidney transplantation
LDKT: Living donor kidney transplantation
