# Supplementary material for: Development and outcomes of surgical and urological kidney transplantation programs in Germany: a total population analysis from 2006 to 2021
Source: World J Urol. 2024 Feb 1;42(1):65. doi: 10.1007/s00345-023-04740-1 (PMC10834564; doi:10.1007/s00345-023-04740-1)
Supplement: Supplementary file 2 — Supplementary file2 (DOCX 17 KB) [file 345_2023_4740_MOESM2_ESM.docx]

Supplementary Figure 2 Good transplant quality at discharge (DDKTs and LDKTs).
