# Supplementary material for: Development and outcomes of surgical and urological kidney transplantation programs in Germany: a total population analysis from 2006 to 2021
Source: World J Urol. 2024 Feb 1;42(1):65. doi: 10.1007/s00345-023-04740-1 (PMC10834564; doi:10.1007/s00345-023-04740-1)
Supplement: Supplementary file 3 — Supplementary file3 (DOCX 15 KB) [file 345_2023_4740_MOESM3_ESM.docx]

Supplementary Figure 3 Good transplant quality after 1 year. Due to insufficient reporting, only deceased donor transplantations from 2015-2019 are shown.
