# Supplementary material for: Development and outcomes of surgical and urological kidney transplantation programs in Germany: a total population analysis from 2006 to 2021
Source: World J Urol. 2024 Feb 1;42(1):65. doi: 10.1007/s00345-023-04740-1 (PMC10834564; doi:10.1007/s00345-023-04740-1)
Supplement: Supplementary file 4 — Supplementary file4 (DOCX 20 KB) [file 345_2023_4740_MOESM4_ESM.docx]

**Supplementary Tables**

| Year | complications  urology;  n (%) | procedures urology; n | complications  surgery;  n (%) | procedures  surgery; n | p-value  (chi^2^) |
| --- | --- | --- | --- | --- | --- |
| 2013 | 142 (25,8) | 550 | 245 (19,6) | 1298 | 0,003 |
| 2014 | 120 (20,8) | 577 | 242 (19,3) | 1252 | 0,464 |
| 2015 | 82 (15,7) | 522 | 267 (19,5) | 1367 | 0,056 |
| 2016 | 92 (16,6) | 555 | 206 (18,1) | 1137 | 0,435 |
| 2017 | 104 (20,2) | 519 | 214 (19,0) | 1127 | 0,549 |
| 2018 | 97 (16,0) | 607 | 253 (18,4) | 1376 | 0,198 |
| 2019 | 85 (15,6) | 546 | 268 (21,5) | 1244 | 0,004 |
| **All** | **722 (18,6)** | **3876** | **1695 (19,3)** | **8801** | **0,404** |

Supplementary table 1 Intra- and postoperative complications in deceased donor kidney transplants

**a)**

| Year | Immediate function DDKT  Urology; n (%) | procedures urology; n | Immediate function DDKT  Surgery; n (%) | procedures  surgery; n | p-value  (chi^2^) |
| --- | --- | --- | --- | --- | --- |
| 2013 | 307 (68,9) | 445 | 700 (78,4) | 893 | <0,001 |
| 2014 | 311 (76,8) | 405 | 718 (84,9) | 846 | 0,001 |
| 2015 | 346 (74,3) | 466 | 731 (75,9) | 963 | 0,495 |
| 2016 | 283 (72,2) | 392 | 709 (87,3) | 812 | <0,001 |
| 2017 | 286 (68,9) | 414 | 653 (77,7) | 840 | 0,001 |
| 2018 | 357 (74,4) | 480 | 749 (79,3) | 944 | 0,033 |
| 2019 | n.a. |  | n.a. |  |  |
| 2020 | 263 (70,3) | 374 | 658 (78,1) | 843 | 0,004 |
| **All** | **2153 (72,3)** | **2976** | **4918 (80,1)** | **6141** | **<0,001** |

**b)**

| Year | Immediate function LDKT  Urology; n (%) | procedures urology; n | Immediate function LDKT  Surgery; n (%) | procedures  surgery; n | p-value  (chi^2^) |
| --- | --- | --- | --- | --- | --- |
| 2013 | 216 (96,4) | 224 | 458 (96,1) | 477 | 0,792 |
| 2014 | 176 (94,5) | 186 | 388 (97,0) | 400 | 0,159 |
| 2015 | 165 (99,4) | 166 | 443 (98,9) | 448 | 0,566 |
| 2016 | 156 (91,8) | 170 | 377 (95,9) | 393 | 0,043 |
| 2017 | 147 (96,1) | 153 | 378 (94,7) | 399 | 0,513 |
| 2018 | 163 (97,6) | 167 | 422 (97,7) | 432 | 0,953 |
| 2019 | n.a. |  | n.a. |  |  |
| 2020 | 75 (96,2) | 78 | 330 (100) | 330 | 0,004 |
| **All** | **1098 (96)** | **1144** | **2796 (97,1)** | **2879** | **0,065** |

Supplementary table 2 Immediate function of grafts in DDKTs (a) and LDKTs (b)

**a)**

| Year | Good quality DDKT  Urology; n (%) | procedures urology; n | Good quality DDKT  Surgery; n (%) | procedures  surgery; n | p-value  (chi^2^) |
| --- | --- | --- | --- | --- | --- |
| 2013 | 327 (78,9) | 415 | 738 (85,3) | 865 | 0,004 |
| 2014 | 331 (83,4) | 397 | 801 (86,4) | 927 | 0,151 |
| 2015 | 365 (82,8) | 441 | 802 (85,9) | 934 | 0,134 |
| 2016 | 343 (83,1) | 413 | 761 (79,7) | 954 | 0,158 |
| 2017 | 330 (93,8) | 352 | 694 (85,9) | 808 | <0,001 |
| 2018 | 387 (84,0) | 461 | 830 (86,9) | 955 | 0,133 |
| **All** | **2083 (84,0)** | **2479** | **4671 (85,8)** | **5443** | **0,037** |

**b)**

| Year | Good quality LDKT  Urology; n (%) | procedures urology; n | Good quality LDKT  Surgery; n (%) | procedures  surgery; n | p-value  (chi^2^) |
| --- | --- | --- | --- | --- | --- |
| 2013 | 138 (96,5) | 143 | 537 (96,3) | 558 | 0,880 |
| 2014 | 118 (98,3) | 120 | 449 (94,5) | 475 | 0,079 |
| 2015 | 168 (96,6) | 174 | 456 (95,6) | 477 | 0,589 |
| 2016 | 163 (95,1) | 171 | 378 (95,9) | 394 | 0,738 |
| 2017 | 139 (97,0) | 143 | 379 (94,2) | 402 | 0,166 |
| 2018 | 164 (96,9) | 169 | 455 (97,1) | 469 | 0,986 |
| 2019 | 145 (94,2) | 154 | 346 (96,9) | 357 | 0,140 |
| **All** | **1035 (96,4)** | **1074** | **3000 (95,8)** | **3132** | **0,404** |

Supplementary table 3 Good quality of grafts at the day of discharge for DDKTs (a) and LDKTs (b)

| Year | Good quality 1 year, DDKT  Urology; n (%) | procedures urology; n | Good quality 1 year, DDKT  Surgery; n (%) | procedures  surgery; n | p-value  (chi^2^) |
| --- | --- | --- | --- | --- | --- |
| 2015 | 525 (95,1) | 552 | 1164 (97,4) | 1195 | 0,013 |
| 2016 | 568 (96,3) | 590 | 1177 (95,7) | 1230 | 0,560 |
| 2017 | 517 (95,9) | 539 | 1158 (95,0) | 1219 | 0,400 |
| 2018 | 460 (97,1) | 474 | 1020 (96,3) | 1059 | 0,470 |
| 2019 | 488 (97,6) | 500 | 1044 (96,2) | 1085 | 0,156 |
| **All** | **2558 (96,3)** | **2655** | **5563 (96,1)** | **5788** | **0,602** |

Supplementary table 4 Good quality of grafts in DDKTs 1 year postoperatively
